# Safety and Efficacy of Inclisiran in Hyperlipidemia: An Updated Meta‐Analysis of Randomised Controlled Trials

**DOI:** 10.1002/edm2.70039

**Published:** 2025-03-14

**Authors:** Jawad Basit, Mushood Ahmed, Priyansha Singh, Areeba Ahsan, Eeshal Zulfiqar, Javed Iqbal, Maurish Fatima, Prakash Upreti, Mohammad Hamza, M Chadi Alraies

**Affiliations:** ^1^ Department of Medicine Rawalpindi Medical University Rawalpindi Pakistan; ^2^ Smt. Nathiba Hargovandas Lakhmichand Municipal Medical College Ahmedabad India; ^3^ Department of Medicine Foundation University Medical College Islamabad Pakistan; ^4^ Department of Medicine Dow University of Health Sciences Karachi Pakistan; ^5^ Nursing Department Hamad Medical Corporation Doha Doha Qatar; ^6^ Department of Medicine King Edward Medical University Lahore Pakistan; ^7^ Sands Constellation Heart Institute, Rochester Regional Health Rochester New York USA; ^8^ Department of Hospital Medicine Guthrie Cortland Medical Center Cortland New York USA; ^9^ Cardiovascular Institute Wayne State University/Detroit Medical Center Detroit Michigan USA

**Keywords:** hyperlipidemia, Inclisiran, meta‐analysis

## Abstract

**Introduction:**

Inclisiran, a small interfering RNA (siRNA), reduces the levels of low‐density lipoproteins (LDL) in the body by preventing the hepatic synthesis of proprotein convertase subtilisin/kexin type 9 (PCSK9). However, there is limited pooled data regarding the efficacy and safety of inclisiran in patients with hypercholesterolemia.

**Methods:**

PubMed/MEDLINE, Embase and the Cochrane Library were searched by investigators from inception till July 2024 to identify randomised controlled trials (RCTs) that investigated inclisiran in patients with hypercholesterolemia. Weighted mean differences (MDs) for continuous outcomes and risk ratios (RRs) for the dichotomous outcomes were pooled. The analysis was conducted using the random effects model, and a *p*‐value of < 0.05 was considered statistically significant.

**Results:**

A total of 8 RCTs reporting data for 5016 patients were included in the pooled analysis. Our pooled analysis demonstrated that inclisiran was associated with a significant decline in the % of LDL‐C levels (MD = −50.42, 95% CI: −56.15 to −44.70), % of PCSK9 levels (MD = −78.57, 95% CI: −81.64 to −75.50), % of total cholesterol levels in the body (MD = −31.22, 95% CI: −33.08.15 to −29.37), and apo B levels (MD = −41.47, 95% CI: −44.83 to −38.11) when compared with the control group. The risk of all‐cause death, cardiovascular death, major adverse cardiovascular events, myocardial infarction, stroke, and serious adverse events remained comparable (*p* > 0.05) across the two groups.

**Conclusion:**

Inclisiran reduces LDL‐C, PCSK9, cholesterol and apo‐B levels in the body without increasing the risk of serious adverse events.

## Graphical abstract

Central illustration describing the safety and efficacy of inclisiran in hyperlipidemias.

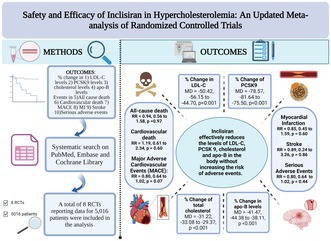

## 1 Introduction

Atherosclerotic vascular disease (ASCVD) is the primary cause of mortality and disability globally, and this status is predicted to persist beyond 2040 [1]. The complex nature of ASCVD necessitates preventive behaviour, healthy lifestyles, and managing risk factors like blood pressure and lipid levels [2]. Research and comprehensive analyses have established a direct connection between elevated levels of low‐density lipoprotein cholesterol (LDL‐C) and ASCVD [3]. In contrast to the clinical evidence, the real‐world data reports that 80% of ASCVD patients fall short of achieving their LDL‐C goals due to challenges such as ‘therapeutic inertia’ (a term used to describe the reluctance to initiate or intensify therapy), medication nonadherence and drug side effects [4, 5, 6, 7].

Statins, despite being the drug of choice for ASCVD prevention and treatment, only 20%–40% of exceptionally high‐risk patients who receive statin monotherapy go on to meet the recommended reduced LDL cholesterol levels. Individuals who do not meet their objectives need to use combination therapies [8, 9]. The emerging non‐statin lipid‐lowering therapies include ezetimibe, bempedoic acid and PCSK9 inhibitors. Two approaches are being globally used by the injectable therapies directed against PCSK9. Firstly, monoclonal antibodies targeting PCSK9 within the bloodstream have demonstrated efficacy in lowering the LDL‐C and cholesterol levels and reducing the incidence of cardiovascular events [10, 11]. Alternatively, inclisiran, a small interfering ribonucleic acid (siRNA), has been approved by the United States Food and Drug Administration (US FDA) and the European Medicines Agency (EMA) [12, 13].

Utilising RNA interference and associated RNA silencing pathways offers the chance to control gene expression through a highly specialised endogenous method [14]. Inclisiran is a chemically synthesised siRNA that effectively induces PCSK9‐specific RNA silencing in hepatocytes [15]. Additionally, it is administered twice yearly with characteristics of well tolerance, rapid and sustained reduction in LDL‐C, and better attainment of LDL‐C goal than other therapeutic drugs [16, 17, 18]. Although prior meta‐analyses [19, 20] have examined inclisiran's effectiveness in treating individuals with hypercholesterolemia, the investigators included studies with short‐term follow‐up periods. Long‐term follow‐ups of ORION trials and new studies have been published which warrant the need for an updated meta‐analysis with enhanced statistical power. Our meta‐analysis has pooled all the latest and up‐to‐date data by conducting a comprehensive study exploring the clinical outcomes of inclisiran.

## 2 Methods

The systematic review and meta‐analysis was done using the guidelines established by the Preferred Reporting Items for Systematic Review and Meta‐Analysis (PRISMA) [21].

### 2.1 Data Sources and Search Strategy

Two authors independently searched PubMed/MEDLINE, EMBASE, Cochrane Library, and ClinicalTrials.gov to identify trials that explored the effects of inclisiran in patients with hypercholesterolemia, compared to standard care or placebo. The search covered all publications in these databases from their inception until July 2024, with no language restrictions. The reviewers thoroughly examined references from the trials that were retrieved, as well as from previous meta‐analyses and review articles, to verify that all pertinent studies were incorporated. The selected studies were imported to EndNote X9 (Clavirate Analytics) to identify and remove duplicates. To maintain the credibility and validity of the research, this meta‐analysis exclusively included the study with the highest number of participants in instances where multiple studies used the same set of patients. The medical subject heading (MeSH) terms employed included ‘Inclisiran,’ ‘hyperlipidemia’, ‘cholesterol’, ‘RCT’ and ‘LDL’. The details of the search strings used are provided in Table S1.

### 2.2 Study Selection

The studies were eligible if they: (i) were randomised controlled trials (RCT) and had a follow‐up of at least 2 weeks; (ii) included patients with hypercholesterolaemia; (iii) had adult male or female participants who were at least 18 years old; (iv) compared inclisiran to either standard care or placebo; (v) evaluated at least one of the predetermined efficacy and safety endpoints. The efficacy endpoints included changes in LDL‐C levels, % of PCSK9, cholesterol levels, apo‐B levels, all‐cause mortality, cardiovascular mortality and cardiovascular events. The safety outcomes were adverse events.

### 2.3 Data Extraction and Quality Assessment

Two authors independently reviewed trials based on their titles and abstracts per the inclusion criteria. The full texts of the articles were then examined, and any trials that reported one or more predetermined outcomes were included. A third author was consulted in case of a disagreement. The following data was extracted for each study: author surname, year, mean patient age, dose of treatment, cardiovascular risk factor (hypertension, diabetes, previous ASCVD), additional therapies used, level of PCSK9 and mean duration of follow‐up. This study included randomised controlled trials, so Version 2 of the Cochrane tool was used to assess the risk of bias assessment [22].

### 2.4 Statistical Analysis

Using RevMan, Version 5.4 (Nordic Cochrane Center, Copenhagen, Denmark), the data was statistically analysed. The findings were displayed in terms of relative risk (RR) for the dichotomous results and weighted mean difference (WMD) for the continuous outcomes, each with 95% confidence intervals (CIs). The effect sizes were pooled using the DerSimonian Laird random‐effects model [23], while the results of the pooled analysis were visualised using forest plots. The trials were evaluated for heterogeneity using the Higgins I2 test, with the categorisation being as follows: a value of less than 25% indicates a low risk; 25%–75% indicates a moderate risk; and > 75% indicates a high risk [24]. Publication bias could not be assessed using funnel plots or statistical tests as the pooled studies were < 10. In all cases, a *p*‐value < 0.05 was considered statistically significant.

## 3 Results

### 3.1 Study Selection

A systematic literature search yielded 1143 records. The duplicate studies were removed, and two investigators performed primary screening based on study titles and abstracts. This was followed by secondary screening in which full texts of studies were reviewed by the researchers. A total of 8 RCTs [17, 25, 26, 27, 28, 29, 30, 31] met the inclusion criteria and were included in the pooled analysis. The PRISMA flowchart (Figure S1) depicts the study selection process.

**FIGURE 1:**
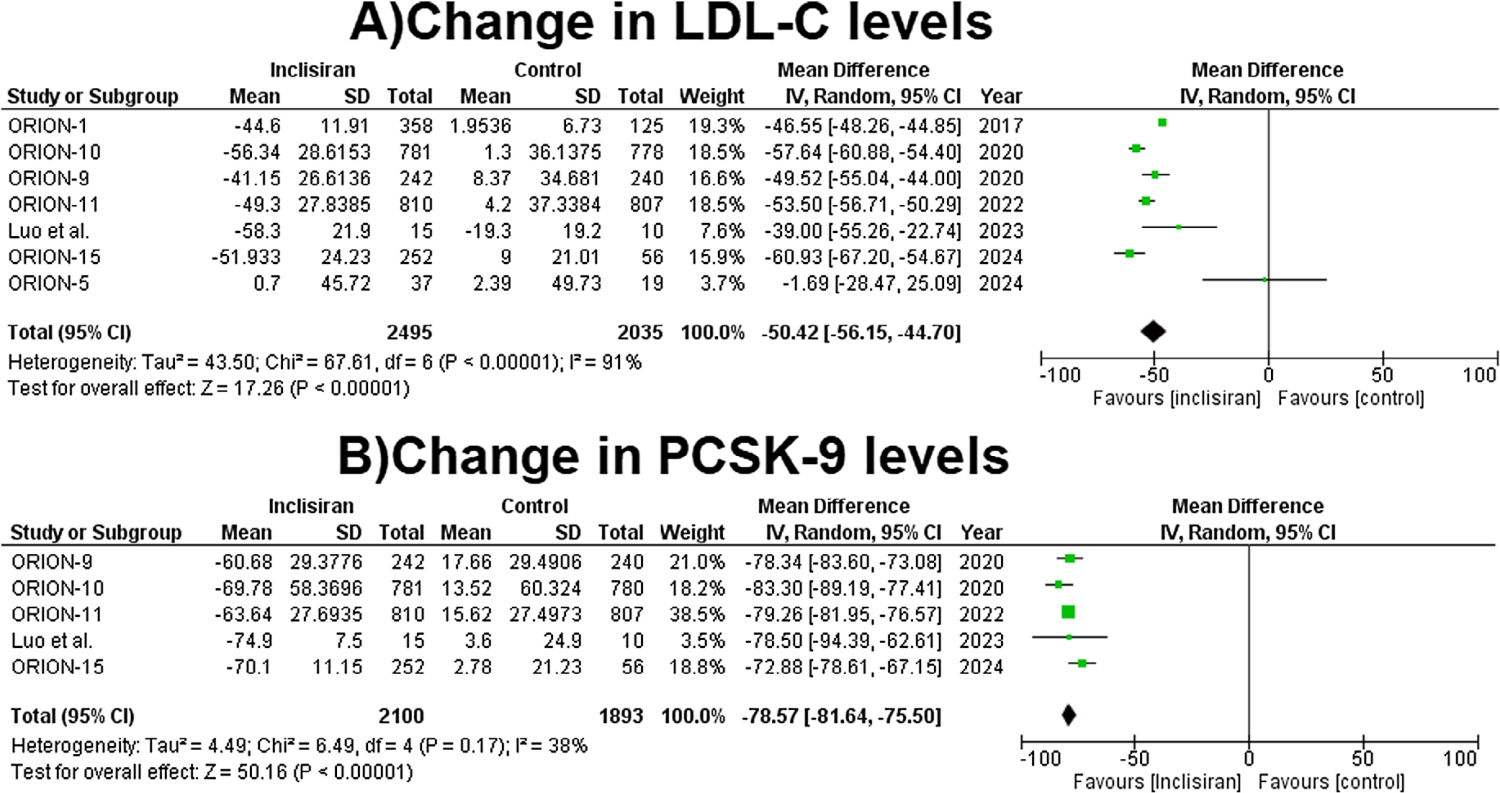
Forest plots for (A) change in LDL‐C levels and (B) change in PCSK‐9 levels.

### 3.2 Study Characteristics

A total of 8 RCTS with 5016 participants were included in this study, 2751 in the inclisiran group and 2265 in the placebo group. Among these trials, ORION‐5, 9, 10, 11 and VICTORIAN‐INITIATE were phase 3 trials and ORION‐1 and ORION‐15 were phase 2 trials. ORION‐14 and ORION‐15 were the earliest clinical trials to evaluate the efficacy and safety of inclisiran in Chinese and Japanese patients, respectively. All of the included studies had a primary endpoint of change in LDL‐C level from baseline and were double‐blinded placebo‐controlled trials.

ORION‐1 included patients with cardiovascular disease (CVD) or equivalent risk with increased LDL‐C levels. The study tested both single and double doses of inclisiran at varying doses: 100, 200, 300 and 500 mg. ORION‐5 included participants with a diagnosis of homozygous familial hypercholesterolemia (HoFH) if they had a history of untreated LDL‐C levels exceeding 500 mg/dL, developed xanthomas at an early age, or had evidence of heterozygous familial hypercholesterolemia in both parents. ORION‐5 and ORION‐9 used 300 mg of inclisiran. In the ORION‐9, ORION‐10, and ORION‐11 trials, patients with CVD or similar high CVD risk, along with LDL‐C levels higher than 70 mg/dL, were admitted. Specifically, ORION‐9 focused on individuals with heterozygous familial hypercholesterolemia who had LDL‐C levels exceeding 100 mg/dL. ORION‐14 included patients with an LDL‐C level at screening higher than 100 mg/dL and triglycerides of no more than 400 mg/dL. ORION‐15 included patients with hypercholesterolemia, including heterozygous familial hypercholesterolemia (HeFH), and evaluated both single and double doses of inclisiran at varying dosages of 100, 200, and 300 mg. The VICTORIAN‐INITIATE trial included adults with a past diagnosis of atherosclerotic cardiovascular disease (ASCVD). Eligible participants had LDL‐C levels of 70 mg/dL or higher, non‐HDL‐C levels of 100 mg/dL or more, and fasting triglycerides below 500 mg/dL. Detailed baseline characteristics are presented in Table 1; Table S2.

**TABLE 1:** Baseline characteristics of included studies.

Trial	Publication Year	Sample size		Follow up	Dose of treatment	Age‐ mean ± SD****		Males‐no** (%)		Hypertension‐no** (%)		Diabetes‐no** (%)		Smoking‐no** (%)	
		Inclisiran	Placebo			Inclisiran	Placebo	Inclisiran	Placebo	Inclisiran	Placebo	Inclisiran	Placebo	Inclisiran	Placebo
ORION‐1	2019	370	127	360 days	Patients were randomly assigned to receive a single dose of placebo or 200, 300 or 500 mg* of inclisiran or two doses (at Days 1 and 90) of placebo or 100, 200, or 300 mg* of inclisiran	64.1 ± 9.4	62.8 ± 10.3	45 (74)	33 (53.2)	43/61 (70.5)	44/61 (71.0)	14 (23)	14 (23)	7 (11.5)	8 (12.9)
ORION‐3	2023	277	92	4 years	Inclisiran sodium was administered as a single dose of 200, 300 or 500 mg on day 1 or two doses of 100 mg, 200 mg, or 300 mg on Day 1 and 90	63·3 ± 11·1	61·9 ± 10·6	188/290 (65%)	55/92 (60)	187/283 (66)	61/90 (68)	69/283 (24)	18/89 (20)	NR***	NR***
ORION‐5	2024	37	19	150 days	300 mg of inclisiran sodium (equivalent to 284 mg* of inclisiran)	43.8 ± 13.4	40.7 ± 12.1	female: 23 (62.2)	11 (57.9)	15 (40.5)	6 (31.6)	2 (5.4)	1 (5.3)	4 (10.8)	2 (10.5)
ORION‐9	2020	242	240	540 days	300 mg*	56 (47–63)*	56 (46–64)*	112 (46.3)	115 (47.9)	102 (42.1)	101 (42.1)	20 (8.3)	28 (11.7)	28 (11.6)	28 (11.7)
ORION‐10	2020	781	780	540 days	284 mg*	66.4 ± 8.9	65.7 ± 8.9	535 (68.5)	548 (70.3)	714 (91.4)	701 (89.9)	371 (47.5)	331 (42.4)	123 (15.7)	111 (14.2)
ORION‐11	2022	811	807	540 days	285 mg*	64.8 ± 8.3	64.8 ± 8.7	579 (71.5)	581 (72.0)	640 (79.0)	661 (81.9)	296 (36.5)	272 (33.7)	160 (19.8)	132 (16.4)
ORION‐14	2023	30	10	90 days	A single dose of either inclisiran sodium 100 or 300 mg* s.c. injection	59.5 ± 7.45	57.3 ± 9.59	3 (20.0%)	4 (40.0)	8 (53.3)	3 (30.0)	5 (33.3)	1 (10.0)	NR***	NR***
ORION‐15	2024	255	57	360 days	Inclisiran sodium 100, 200, or 300 mg*, or placebo and dosed subcutaneously on Days 1, 90, and 270	63.0 ± 10.7	63.8 ± 11.1	22 (22.2)	18 (31.6)	68 (68.7)	40 (70.2)	60 (60.6)	31 (54.4)	20 (20.2)	8 (14.0)
VICTORIAN‐INITIATE	2024	225	225	270 days	284 mg*	66 (35, 87)*	68 (27, 89)*	158 (70.2)	153 (68.0)	201 (89.3)	209 (92.9)	95 (42.2)	95 (42.2)	NR***	NR***

Abbreviations: mg*, milligram, no**, number, NR***, not reported, SD****, standard deviation.

The quality assessment of the included trials showed some concerns in 2 RCTs, mainly related to missing outcomes data (Figures S2 and S3).

### 3.3 Efficacy Outcomes

#### 3.3.1 Change in LDL‐C Levels

The use of inclisiran was associated with a significant reduction in the % of LDL‐C levels as compared to the control group (MD = −50.42, 95% CI: −56.15 to −44.70, *p* = < 0.00001, *I* ^2^ = 91%, Figure 1A).

#### 3.3.2 Change in % of PCSK9

Compared to the control group, the use of inclisiran significantly reduced the % of PCSK9 levels (MD = −78.57, 95% CI: −81.64 to −75.50, *p* = < 0.00001, *I* ^2^ = 38%, Figure 1B).

#### 3.3.3 Change in Cholesterol Levels

**FIGURE 2:**
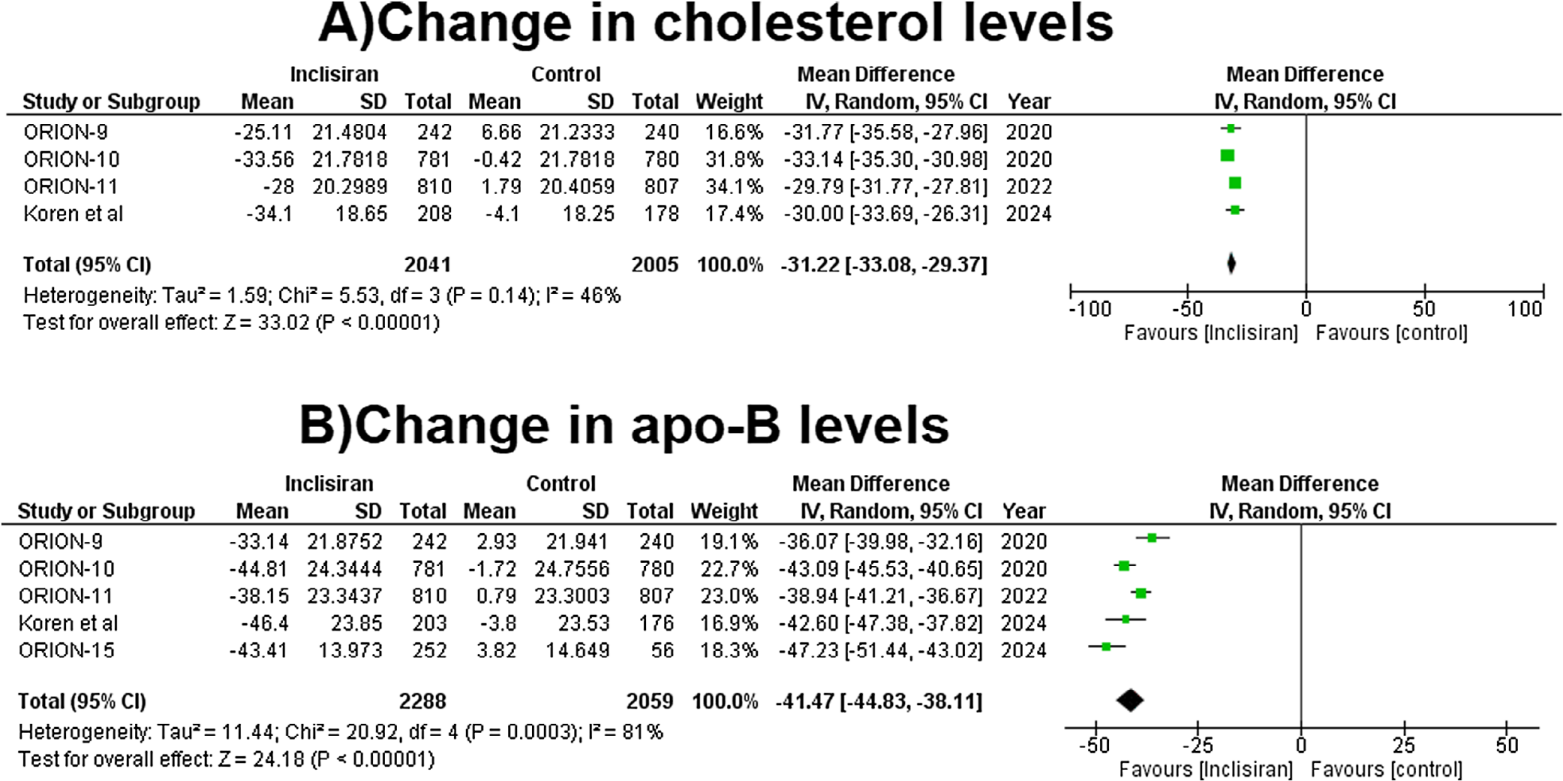
Forest plots for (A) change in cholesterol levels and (B) change in apo‐B levels.

The use of inclisiran significantly reduced the % of total cholesterol levels when compared to the control group (MD = −31.22, 95% CI: −33.08.15 to −29.37, *p* = < 0.00001, *I* ^2^ = 46%, Figure 2A).

#### 3.3.4 Change in Apo‐B Levels

The pooled analysis showed a significant decline in the apo B levels with the use of inclisiran as compared to the control group (MD = −41.47, 95% CI: −44.83 to −38.11, *p* = < 0.00001, *I* ^2^ = 81%, Figure 2B).

#### 3.3.5 Cardiovascular Events

**FIGURE 3:**
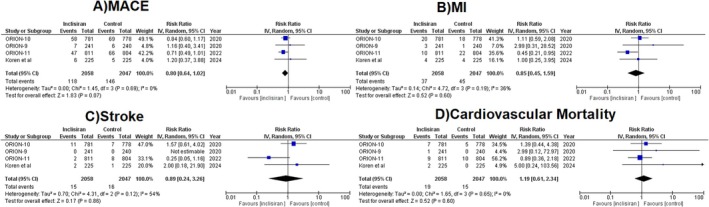
Forest plots for (A) MACE, (B) MI, (C) Stroke and (D) cardiovascular mortality.

Our pooled analysis demonstrated nonsignificant differences in the risk of major adverse cardiovascular events (RR = 0.80, 95% CI: 0.64–1.02, *p* = 0.07, *I* ^2^ = 0%, Figure 3A), myocardial infarction (RR = 0.85, 95% CI: 0.45–1.59, *p* = 0.60, *I* ^2^ = 36%, Figure 3B), stroke (RR = 0.89, 95% CI: 0.24–3.26, *p* = 0.86, *I* ^2^ = 54%, Figure 3C), and cardiovascular mortality (RR = 1.19, 95% CI: 0.61–2.34, *p* = 0.60, *I* ^2^ = 0%, Figure 3D) across the two groups.

#### 3.3.6 All‐Cause Mortality

The pooled analysis showed that the risk of all‐cause mortality (RR = 0.94, 95% CI: 0.56–1.58, *p* = 0.83, *I* ^2^ = 0%, Figure S4) remained comparable across the two groups.

### 3.4 Safety Outcomes

#### 3.4.1 Adverse Events

The pooled analysis demonstrated a significantly increased risk of total adverse events in the inclisiran group compared to the control group (RR = 1.06, 95% CI: 1.01–1.11, *p* = 0.02, *I* ^2^ = 17%, Figure S5). But this was mainly driven by non‐serious adverse events observed in the inclisiran group (RR = 1.08, 95% CI: 0.98–1.19, *p* = 0.11, *I* ^2^ = 45%, Figure S6) as the risk of serious adverse events was comparable across the two groups (RR = 0.90, 95% CI: 0.72–1.12, *p* = 0.36, *I* ^2^ = 50%, Figure S7). However, the risk of injection site reaction (RR = 6.45, 95% CI: 2.41–17.26, *p* = 0.0002, *I* ^2^ = 55%, Figure S8) was significantly increased in the inclisiran group as compared to the control group.

## 4 Discussion

In this comprehensive meta‐analysis, which encompassed 8 RCTs, we evaluated the effectiveness and safety profile of inclisiran in hyperlipidemia. Our pooled analysis found a significant decline in lipid profiles, including changes in LDL‐c levels, percentage of PCSK9, cholesterol, and apo‐B. However, the risk of all‐cause and cardiovascular mortality, cardiac events, and stroke were comparable between the two groups. Regarding safety outcomes, there was an increased risk of total adverse events and injection site reactions with inclisiran.

Since LDL‐C significantly contributes to ASCVD risk, achieving and maintaining target levels is paramount [32]. A substantial number of high‐risk patients on statins have been shown to discontinue the treatment either due to inconvenience from the high frequency of its intake or symptoms of myalgia or myopathy [33, 34, 35]. Hence, due to its efficacy, inclisiran addresses the dual obstacles of high cholesterol: the requirement for additional lowering of LDL‐C levels and the challenge of low compliance. Furthermore, inclisiran is a brief synthetic siRNA that functions as a guide, capable of bonding with the corresponding mRNA of PCSK9 and prompting its degradation [32]. This inhibition causes a decrease in intra‐and extra‐cellular PCSK9 protein levels, resulting in an effective and durable reduction in LDL levels. Additionally, compared to monoclonal antibodies against PCSK9, which require frequent injections, inclisiran has a twice‐yearly dosing regimen, which can improve its adherence rate and provide a long‐term reduction in cardiovascular events [36].

Inclisiran has a unique structural alteration that results in extended biological activity and improved stability. It has also benefited patients with liver and renal impairments, with no siRNA‐induced peripheral neuropathy [32, 37, 38]. The combined safety analysis showed similar outcomes in both groups for all safety measures, except for a notably higher risk of total adverse events in the intervention group. Wang et al. reported a greater risk of TEAE in their systematic review. However, this could be attributed to their small sample size and the fact that the study was conducted in the early phase of drug development [33]. While the incidence of TEAEs was attributed to non‐serious adverse events, none of the participants had discontinued the drug due to the adverse effects [15].

Although inclisiran reduced the LDL‐C levels, it did not lead to a significantly reduced risk of cardiovascular events in our pooled analysis. Hence, other PCSK‐9 inhibitors that have proven cardiovascular advantages are currently preferred over inclisiran for lowering LDL‐C levels in patients not responding to statin therapy or requiring additional LDL‐C‐lowering treatment options. Similarly, ezetimibe (cholesterol absorption inhibitor) is preferred over inclisiran by physicians in patients with hyperlipidaemia [39]. This is driven by the low cost of ezetimibe and its established cardiovascular benefits. The role of inclisiran in patients with ASCVD will become clear as studies report its long‐term cardiovascular benefits. The ORION‐4 trial is expected to enrol 15,000 patients with ASCVD along with a follow‐up of 5 years to investigate the impact of inclisiran on MACE. However, the trial results will be available around 2026 [40]. Considering these factors, inclisiran can be used as an alternative for PCSK‐9 inhibition in patients with intolerance to ezetimibe, alirocumab or evolocumab [41].

The cost‐effectiveness of inclisiran has been debated whether the drug is affordable for patients for its added benefits versus standard care. A study by Desai et al. evaluated the cost‐effectiveness of inclisiran in U.S. patients with atherosclerotic cardiovascular disease [42]. Inclisiran was found to be cost‐effective at annual prices of $6383, $9973, and $13,563 for willingness‐to‐pay thresholds of $50,000, $100,000 and $150,000 per quality‐adjusted life year (QALY), respectively. At its current price of $3250 per dose ($6500 per year), inclisiran's incremental cost‐effectiveness ratio was $51,686, above the $50,000/QALY threshold. Other studies have also reported a high cost of inclisiran compared to other PCSK9 inhibitors [43, 44]. These findings highlight the need for optimised pricing of inclisiran for its wider adoption in a clinical perspective.

There were several limitations to our study. Due to wide variations in dose across the studies, sub‐group analyses could not be performed. The ORION trials had non‐systematic reporting of serious adverse events, which could have compromised the reliability of the evidence. Also, a larger sample size research with extended follow‐up can further clarify the status of serious side effects. We could not assess the publication bias due to fewer studies (< 10). Additionally, some outcomes had a high level of heterogeneity.

## 5 Conclusion

Inclisiran is a well‐tolerated and efficacious drug for hypercholesterolemia. The intervention substantially reduces lipid parameters like LDL‐C, PCSK9, Apo‐B and total cholesterol without increasing the risk of major adverse events. However, more trials are required to assess its impact on reducing MACE.

## Author Contributions

**Jawad Basit** and **Mushood Ahmed:** conceptualization, data curation and project administration. **Jawad Basit**, **Mushood Ahmed**, **Javed Iqbal** and **M Chadi Alraies:** supervision. **Mushood Ahmed** and **Priyansha Singh:** formal analysis of data. **Mushood Ahmed**, **Areeba Ahsan**, **Javed Iqbal** and **Eeshal Zulfiqar:** formal analysis, methodology and software. **Areeba Ahsan** and **Eeshal Zulfiqar:** writing the original draft. **Mohammad Hamza** and **Prakash Upreti**, **M Chadi Alraies** and **Jawad Basit:** writing, reviewing and editing. **Prakash Upreti** and **Priyansha Singh:** visualisation and validation. **Maurish Fatima:** visualization, writing review and editing.

## Ethics Statement

The authors have nothing to report.

## Consent

The authors have nothing to report.

## Conflicts of Interest

The authors declare no conflicts of interest.

## Supporting information


Appendix S1.

## Data Availability

All data generated or analysed during this study are included in this article. Further inquiries can be directed to the corresponding author.
