# Supplementary material for: Safety and Efficacy of Inclisiran in Hyperlipidemia: An Updated Meta‐Analysis of Randomised Controlled Trials
Source: Endocrinol Diabetes Metab. 2025 Mar 14;8(2):e70039. doi: 10.1002/edm2.70039 (PMC11907238; doi:10.1002/edm2.70039)

**Supplementary Appendix**

**Table S1:** Search Strategies for Included studies

**Figure S1:** PRISMA flowchart demonstrating the study screening and selection process

**Table S2:** Detailed baseline characteristics of included studies

**Figure S2:** Bias assessment summary for the included trials

**Figure S3:** Bias assessment graph for the included trials

**Figure S4:** Forest plot for all-cause mortality

**Figure S5:** Forest plot for total adverse events

**Figure S6:** Forest plot for non-serious adverse events

**Figure S7:** Forest plot for serious adverse events

**Figure S8**: Forest plot for injection site reaction

Table S1: Detailed search strategies for each database/register

| **Database/Register** | **Search String** | **Records** |
| --- | --- | --- |
| PubMed/MEDLINE | ("aln pcs"[Supplementary Concept] OR "aln pcs"[All Fields] OR "inclisiran"[All Fields] OR "ALN-PCSsc"[All Fields] OR "PCSK9 siRNA"[All Fields] OR ("pcsk9 targeting"[All Fields] AND ("rna, small interfering"[MeSH Terms] OR ("rna"[All Fields] AND "small"[All Fields] AND "interfering"[All Fields]) OR "small interfering rna"[All Fields] OR "sirna"[All Fields] OR "sirna s"[All Fields] OR "sirnas"[All Fields])) OR ("pcsk9 specific"[All Fields] AND ("rna, small interfering"[MeSH Terms] OR ("rna"[All Fields] AND "small"[All Fields] AND "interfering"[All Fields]) OR "small interfering rna"[All Fields] OR "sirna"[All Fields] OR "sirna s"[All Fields] OR "sirnas"[All Fields]))) AND ("hyperlipidaemia"[All Fields] OR "hyperlipidemias"[MeSH Terms] OR "hyperlipidemias"[All Fields] OR "hyperlipidemia"[All Fields] OR "hyperlipidaemias"[All Fields] OR ("hypercholesterolaemia"[All Fields] OR "hypercholesterolemia"[MeSH Terms] OR "hypercholesterolemia"[All Fields] OR "hypercholesterolaemias"[All Fields] OR "hypercholesterolemias"[All Fields]) OR "high cholesterol"[All Fields] OR "lipid disorder"[All Fields] OR "dyslipidemia"[All Fields] OR "elevated LDL"[All Fields] OR "elevated cholesterol"[All Fields] OR "high LDL"[All Fields] OR "cholesterol levels"[All Fields] OR "lipid levels"[All Fields]) | 280 |
| Embase | ('inclisiran'/exp OR 'inclisiran' OR 'aln-pcssc' OR 'pcsk9 sirna' OR 'pcsk9-targeting sirna' OR 'pcsk9-specific sirna') AND ('hyperlipidemia'/exp OR hyperlipidemia OR hypercholesterolemia OR 'high cholesterol' OR 'lipid disorder' OR 'dyslipidemia' OR 'elevated ldl' OR 'elevated cholesterol' OR 'high ldl' OR 'cholesterol levels' OR 'lipid levels') | 741 |
| Cochrane Library | (inclisiran OR "ALN-PCSsc" OR "PCSK9 siRNA" OR "PCSK9-targeting siRNA" OR "PCSK9-specific siRNA") AND (hyperlipidemia OR hypercholesterolemia OR "high cholesterol" OR "lipid disorder" OR dyslipidemia OR "elevated LDL" OR "elevated cholesterol" OR "high LDL" OR "cholesterol levels" OR "lipid levels") | 84 |
| ClinicalTrials.gov | (inclisiran OR "ALN-PCSsc" OR "PCSK9 siRNA" OR "PCSK9-specific siRNA") AND (hyperlipidemia OR hypercholesterolemia OR "high cholesterol" OR "lipid disorder" OR dyslipidemia OR "elevated LDL" OR "elevated cholesterol" OR "high LDL" OR "cholesterol levels" OR "lipid levels") | 38 |

Figure S1: PRISMA flowchart demonstrating the study screening and selection process


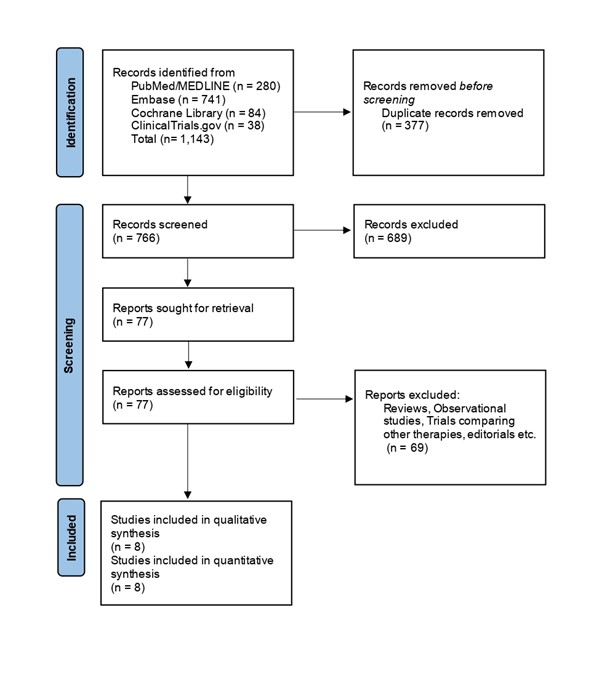


Table S2: Detailed baseline characteristics of included patients

| **Trial** | **Year** | **Previous ASCVD — no./total no. (%)** | | **Statin treatment — no./total no. (%)** | | **Ezetimibe** | | **Cholesterol level — LDL** | | **total cholesterol** | | **apolipoprotein B** | | | **PCSK9, μg/L** | | |
| --- | --- | --- | --- | --- | --- | --- | --- | --- | --- | --- | --- | --- | --- | --- | --- | --- | --- |
|  |  | **Inclisiran** | **Placebo** | **Inclisiran** | **Placebo** | **Inclisiran** | **Placebo** | **Inclisiran** | **Placebo** | **Inclisiran** | **Placebo** | **Inclisiran** | **Placebo** | **Inclisiran** | | **Placebo** |  |
| **ORION-1** | **2019** | 43/61 (70) | 46/62 (74) | 43/59 (73) | 47/61 (77) | 15 (25) | 17 (28) | 131.3±60.3 | 125.2±44.3 | 221.7±65.5 | 208.4±58.7 | 107.4±32.1 | 104.6±31.5 | 416.3±127.3 | | 431.3±132.3 |  |
| **ORION-5** | **2024** | 12 (32.4) | 6 (31.6) | 37 (100.0) | 19 (100.0) | 12 (63.2) | 25 (67.6) | 294.0±136.3 | 356.7±122.4 | 363.0±131.8 | 423.2±122.4 | 192.7±71.8 | 223.4±70.2 | 606.1±447.0 | | 499.2±211.0 |  |
| **ORION-9** | **2020** | NR | NR | 219 (90.5) | 217 (90.4) | 135 (55.8) | 120 (50.0) | 151.4±50.4 | 154.7±58.0 | 230.0±54.6 | 232.4±62.8 | 123.8±33.2 | 124.5±34.8 | 452.2±131.2 | | 429.1±135.3 |  |
| **ORION-10** | **2020** | 781 (100) | 780 (100) | 701 (89.8) | 692 (88.7) | 80 (10.2) | 74 (9.5) | 104.5±39.6 | 104.8±37.0 | 180.6±46.1 | 180.6±43.6 | 94.1±25.6 | 94.6±25.1 | 422.1±176.9 | | 414.9±145.7 |  |
| **ORION-11** | **2022** | 712 (87.9) | 702 (87.0) | 766 (94.6) | 766 (94.9) | 52 (6.3) | 62 (7.7) | 107.2±41.8 | 103.7±36.4 | 187.3±48.2 | 183.3±42.8 | 97.1±28.0 | 95.1±5.2 | 355±98.9 | | 353±97.4 |  |
| **ORION-14** | **2023** | 3 (20.0) | 3 (30.0) | 15 (100.0) | 10 (100.0) | NR | NR | 123 (23.2) | 133 (17.0) | NR | NR | NR | NR | 378 (87.8) | | 319 (48.0) |  |
| **ORION-15** | **2024** | NR | NR | 88 (88.9) | 50 (87.7) | 44 (44.4) | 21 (36.8) | 112.2 (35.50) | 113.1 (32.23) | NR | NR | NR | NR | 400.9 (112.26) | | 422.2 (108.82) |  |
| **VICTORIAN-INITIATE** | **2024** | 211 (93.8) | 218 (96.9) | 203 (90.2) | 202 (89.8) | 1 (0.4) | 4 (1.8) | 97.4 (33.2) | 97.4 (32.4) | 171.8 (40.1) | 171.7 (37.1) | 94.0 (25.3) | 90.9 (24.4) | NR | | NR |  |

ASCVD; Atherosclerotic cardiovascular disease, LDL; low-density lipoproteins,

Figure S2: Bias assessment summary for the included trials


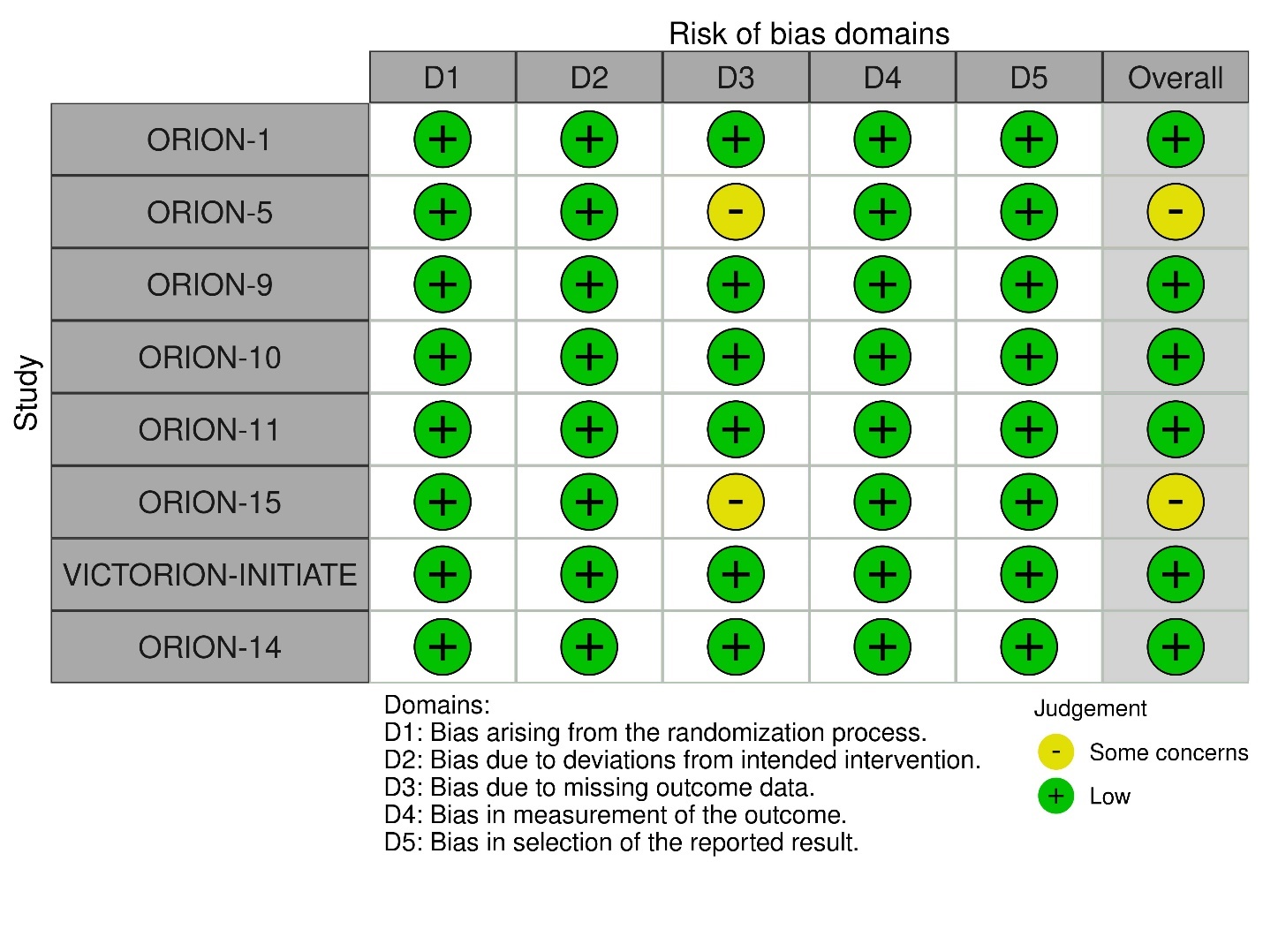


Figure S3: Bias assessment graph for the included trials


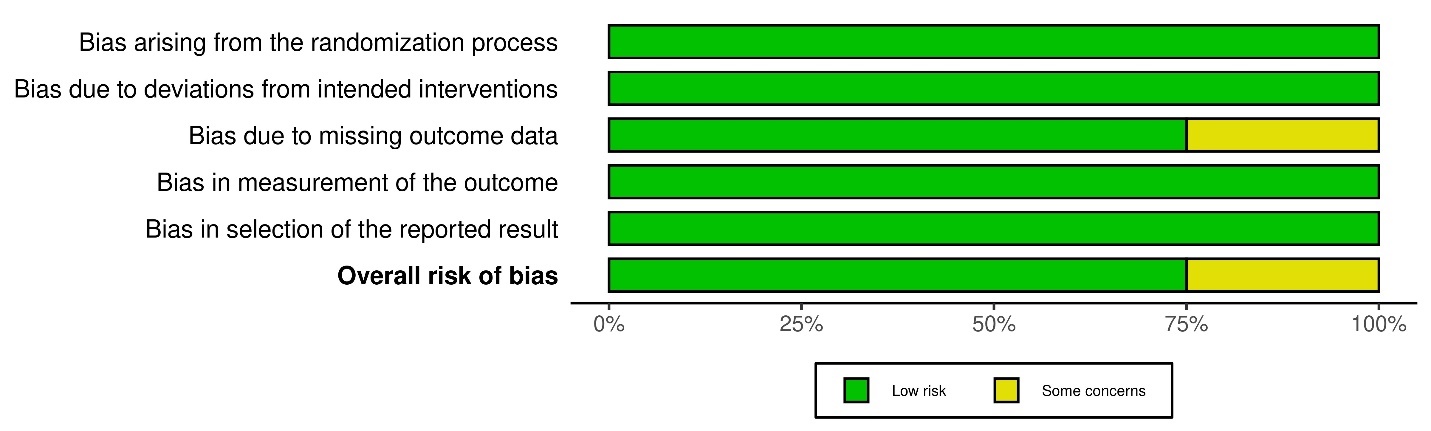


Figure S4: Forest plot for all-cause mortality


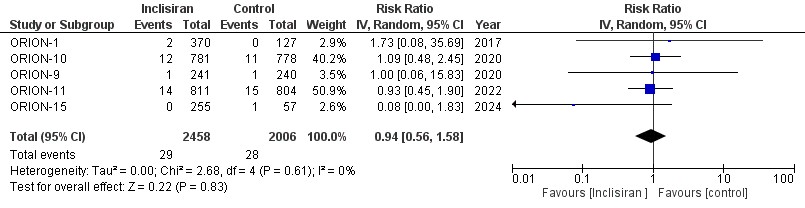


Figure S5: Forest plot for total adverse events


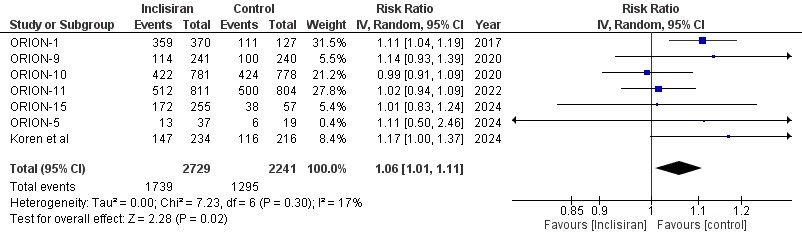


Figure S6: Forest plot for non-serious adverse events


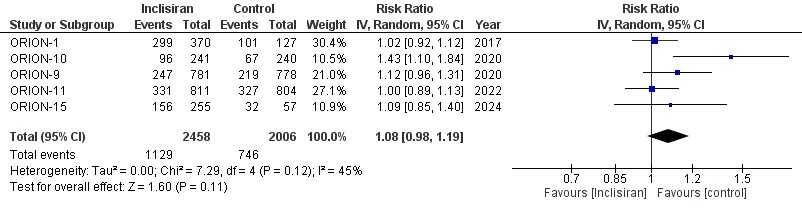


Figure S7: Forest plot for serious adverse events


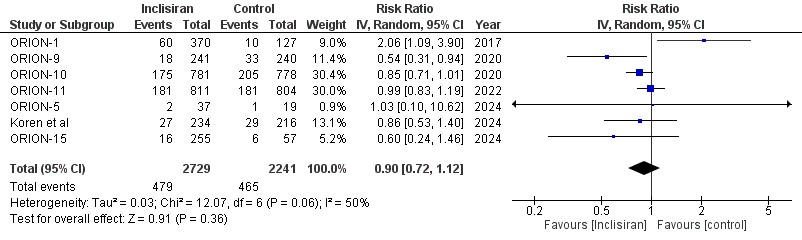


Figure S8: Forest plot for injection site reaction


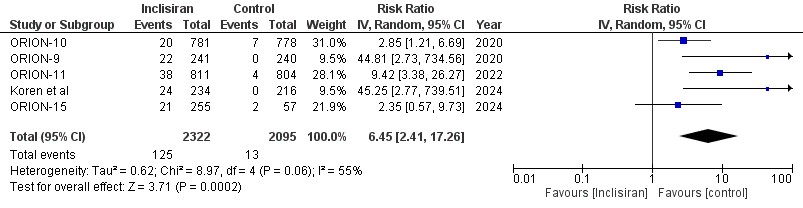

Supplement: Supplementary file 1 — Appendix S1. [file EDM2-8-e70039-s001.docx]
